# The Relationship between Energy Consumption, CO_2_ Emissions, Economic Growth, and Health Indicators

**DOI:** 10.3390/ijerph20032325

**Published:** 2023-01-28

**Authors:** Jing Li, Muhammad Irfan, Sarminah Samad, Basit Ali, Yao Zhang, Daniel Badulescu, Alina Badulescu

**Affiliations:** 1 School of Economics, Minzu University of China, Beijing 100081, China; 2 Department of Economics, COMSATS University Islamabad (C.U.I.), Islamabad 44000, Pakistan; 3 Department of Business Administration, College of Business and Administration, Princess Nourah bint Abdulrahman University, Riyadh 11671, Saudi Arabia; 4 School of Management, Hebei Finance University, Baoding 071051, China; 5 Department of Economics and Business, Faculty of Economic Sciences, University of Oradea, 410087 Oradea, Romania

**Keywords:** life expectancy, mortality rate, CO_2_ emission, cointegration, structural break

## Abstract

The health and wellness of people through life expectancy, mortality rate improvement, and sustaining the productivity of labor contributes a lot to national income. Infrastructure development consumes energy and releases carbon dioxide at different stages of the construction process. The current study explores the nexus between CO_2_ emission, energy consumption, mortality, life expectancy, and GDP in the top five carbon-emitting countries by using time series data from 1975 to 2015. The study used a cointegration technique to find the long- and short-run relationships between study variables. The study also used a structural break test to identify the break time. The results of the correlation matrix show strong positive correlation between CO_2_ emissions and energy consumption. It also reflects a weak correlation with mortality and life expectancy in Japan and Russia. The results of the ADF test indicated that the series are stationary at first difference and provided evidence to use Johansen cointegration test for long- and short-run relationships between independent series. Vector error correction term and ECT method are used to find long-run relationships between cointegrated series and adjustment parameters. For the structural breaks of health indicators and energy consumption study, we used the Gregory Hanson structural break. Mortality rate and life expectancy rate of China, U.S., Russia, India, and Japan show relevant policy changes with economic policies of each country.

## 1. Introduction

In contemporary times, environmental concerns have taken on leading discussions in both emerging and developed economies as a result of environmental degradation. This also raises questions about global warming and climate change, primarily caused by greenhouse gas emissions, sometimes linked to natural causes (i.e., continental drifts, volcanic activity, and solar radiation and ocean currents) and direct and indirect human activity that affect the global atmospheric make-up and the variability of the environment. The researchers have, nevertheless, argued that the acceleration of human activities as a result of the advent of industrialization, the growth of the global population and the need to address such transformations are the principal causes of climate change. Furthermore, human activities such as agricultural and commercial deforestation, fossil fuel combustion, and land-use changes due to population growth contribute significantly to an increase in greenhouse gas emissions [1].

Therefore, Yildirim [2] noted that insufficient energy would have a negative effect on economic performance in various sectors such as transport and the social life of a nation. The rise in energy demand, however, represents a challenge to the global environment. This has led to longer drought, rising sea levels, and the emergence of heatwaves that have serious negative environmental impacts. While the effects of human activity are known, Urry [3] found that greenhouse gas emissions, such as carbon dioxide (CO_2_), have increased into the atmosphere.

The relationship between economic development, nonrenewable energy, and CO_2_ emissions, which is essential to the understanding and refinement of development models in developing countries, has been highlighted in several research studies. Fossil imports and carbon dioxide emissions can be effectively mitigated by societies that have contributed abundant natural resources. Ajmi, Hammoudeh [4] stated that the energy strategy’s implementation is validated to reduce dependence on nonrenewable energy sources. The energy mix is still strongly influenced by nonrenewable energy sources. This explains the long-term sustainability of both energy sources, such as renewables and non-renewables. Emissions of carbon dioxide have increased since 1960 due to coal, oil, gas, and fossil fuels. The use of fossil fuels for transport generates 14% of CO_2_ emissions (IPCC, 2007). According to the National Oceanic and Atmospheric Administration (NOAA), the US, Europe, and China are the main producers of carbon dioxide.

The relationship between health and environment is a prolonged story by default. Health indicators and environmental factors depend on each other by nature and are natural in their functions. Sinha [5] studied India’s causal relationship between carbon emission and mortality rates. An increase in CO_2_ emissions causes mortality rates to rise. To run the industrial sector, human force is a compulsory input. Shortage of human capital for a long time can cause a decline in the export sector in any country.

The Organization [6] investigated CO_2_ consumption, which, nowadays, is critical for human life in the form of electricity and heating. Consumption of energy from fossil fuels has alarming impacts on health indicators.

To the best of our knowledge, no other studies have used CO_2_ emissions, energy consumption, fossil fuel energy consumption, mortality rate, life expectancy, and GDP to find structural breaks of the selected sample countries. Mostly, studies used CO_2_ emissions with economic growth, health expenditures, and urbanization to find causality between variables [5,7]. The approaches were used for investigations of the perception of ecological constituents of well-being [8,9,10].

The main objective of our research is to test the cointegration relationship between variables using the cointegration equation and to investigate the short-run and long-run relationship of variables. Various researchers have established that environmental pollution is caused in developing countries by the use of nonrenewable energy and economic development. By monitoring the model for energy usage, economic growth, and CO_2_ emissions, this research study will help clarify the difference between early investigations. This study will also investigate the structural breaks of health indicators and environmental variables and review the policies adopted in the sample countries using structural breaks.

A closer convergence of the two fields of science and policy requires a deep relation between climate change and human health. The connections are basically triple. First, the effects of climate change, including heat waves, hurricanes, floods, droughts and fires, altered trends of infectious diseases, air pollution, and food shortages, will raise demand for health services. Second, cobenefits to the environment combine long-term benefits from decreased greenhouse gas emissions (GHG) with tangible and short-term public health benefits [11]. The changes in diets, in particular, reduction in meat intake, changes in modal division and, in particular, a transition from private motorized transport to active mobility are two strong leverage points for integrated prevention and public health policies. Third, healthcare is a large and socio-economic field and is itself a major cause of CO_2_ emissions. Organization of Economic Co-operation and Development (OECD) invested on health care an average of nine percent of GDP [12]. In recent decades, spending on health care has always outpaced economic development, driven by ageing populations, noncommunicable diseases in the population’s life, and rapid medical care developments. Direct emissions are relatively low compared to other industries in the health sector as well as in other service sectors in general. However, emissions across the supply chain, as a result of purchases of healthcare products and services, can represent a substantial proportion of national CO_2_ footprints.

In contemporary times, environmental concerns have taken on leading discussions in both emerging and developed economies due to environmental degradation. This also raises questions about global warming and climate change, primarily caused by greenhouse gas emissions, and sometimes linked to natural causes (i.e., continental drifts, volcanic activity, and solar radiation, and ocean currents) and direct and indirect human activity that affect the global atmospheric make-up and the variability of the environment. Researchers have, nevertheless, argued that the acceleration of human activities as a result of the advent of industrialization, the growth of the global population, and the need to address such transformations are the principal causes of climate change. Furthermore, human activities such as agricultural and commercial deforestation, fossil fuel combustion, and land-use changes due to population growth contribute significantly to an increase in greenhouse gas emissions. Despite industrialization’s contribution to boosting economic growth by increasing the volume of products and services generated, shaping life, and enhancing society, it left us with an issue of growing greenhouse emissions. In the world today, energy demand is rising due to the growing population and urbanization. This is vital to keep pace with the rapid upheavals and transformations in global economies. Energy is central to human life and the global economy’s social, economic, and environmental growth. Without energy consumption, it is likely difficult to generate, deliver, or use mainstream goods. Therefore, Yildirim (2020) noted that insufficient energy would have a negative effect on economic performance in various sectors such as transport and the social life of a nation. The rise in energy demand, however, represents a challenge to the global environment. This has led to longer droughts, rising sea levels, and the emergence of heatwaves that have serious negative environmental impacts. While the effects of human activity are known, Sinha [5] found that greenhouse gas emissions, such as carbon dioxide (CO_2_), have increased into the atmosphere.

Economic growth has often contributed to environmental destruction, mostly as a result of development and industrialization, in both developing and industrialized countries. Any country’s economic growth depends on various factors which can have a negative environmental impact, such as unsustainable extraction of natural resources, contamination of the atmosphere, and climate change. Furthermore, in many countries, the rapid rise in urbanization leads to rapid economic growth, resulting in an increase in energy usage. The main problem facing many countries is the amount of carbon dioxide in the atmosphere which is dramatically rising because of energy use and economic development. Energy sources are fossil fuels such as coal and natural oil and gas, resulting in an increased amount of CO_2_ emissions. This led scientists to argue that CO_2_ emissions are invisible and that their impact would take several years to materialize. Even if the reasons identified for the growth in the overall emissions level include numerous factors, such as population size, energy intensity, economic growth, clean nuclear energy use, fossil fuels use, renewable energy, urbanization, and other air pollutants (PM_10_, PM_2.5_, SO_2_, NO_2_, CO, B(a)P), the aim of this study is to understand the effect of energy inconveniences. In light of the rapid global economic growth that results in increased energy consumption, it is important to consider the relationship between these factors in order to achieve a balance between energy consumption, economic growth, and CO_2_ emissions. Furthermore, it would help to directly resolve the risks (i.e., avoid a 40th century world) posed by global climate change.

## 2. Literature Review

Global health indicators are strongly affected by energy use and CO_2_ emissions. Domen [13] investigated the per capita CO_2_ emissions from cities and the average of those cities in which they are located. Agricultural activities supported 14% in emissions, while the biggest share goes to the industrial sector by supporting 19%. According to Domen [13], per capita emissions were different from the average of cities in US and Japan. Domen [13] also reported that developed nations are the big reason behind the highest CO_2_ emissions. Domen [13] also reported the results of high and low earning income groups and the figures were shocking. GHG emissions were 4.52 tons per annum with Rs. 30,000 income per month, while it was just 1.11 tons with income less than Rs. 30,000. Nejat, Jomehzadeh [14] reviewed the top ten countries producing two-third of the world’s CO_2_ emissions by residential energy use. The U.S. and Japan were the big pillars of the increasing trend of CO_2._

From 1980 to 2013, [15] examined the association between oil consumption, economic growth, and environmental degradation in three Asian countries by applying the Johansen cointegration test to check the relationship between the variables in the study. The results examined showed that unidirectional causality runs from oil consumption to economic growth in China and Japan, while oil consumption in South Korea leads to CO_2_ emissions. From 1992 to 2016, Bhat [16] examined the effect of energy use and economic growth on carbon dioxide by using the Panel ARDL model to verify the relation between the variables of the analysis. The findings reviewed showed that resources, labor, population, per capita income, and nonrenewable energy consumption positively affect CO_2_ emissions.

Between 1975 and 2015, Sulaiman, Muniyan [17] investigated the relation between CO_2_ pollution, energy use, and the economy in Malaysia using the ARDL model. The results of the review showed that economic growth is not affected by energy consumption and CO_2_ emissions, while CO_2_ emissions are positively affected by energy consumption and economic growth. By using the autoregressive vector (VAR) model and the Wald test for causality testing, Chen [18] examined the impact of gasoline energy consumption on economic growth in Cameroon. The projected results showed that there is no long-term relationship between the variables in the sample. There is a bidirectional causal link between gasoline consumption and economic development in Cameroon. The estimated results showed that reducing the consumption of gasoline is not a possible solution to maintaining Cameroon’s economic growth without appropriate and established energy policies.

Many studies have been found on CO_2_ emissions and mortality rates, some on energy consumption and health indicators, while some have studied the use of energy and mortality rates in the perspective of economic growth. Some papers explored the relationship between life expectancy, CO_2_, GDP, and the growth of the country. Many other papers explored the relationship between CO_2_ emissions and health indicators [19], while some have studied the impact of urbanization, income level, life expectancy, energy consumption, and economic growth. Some studies had explored Causality and structural breaks between health and environmental variables [20].

A report on the topic of life expectancy declines in the U.S. Rogers [21] has been published. According to the report, since 1993, American life expectancy is declining drastically without identification of any health disease. In 1993, America suffered high mortality rates due to HIV. The death rate in America has increased by 1.2% in 2015 with respect to 2014 [22]. What policy measures are taken from America after this decline and how those policies would be useful is the concern of our study. The following study represented a 0.20% increase in carbon emission with an increase of 1% urbanization process. Khalid, Dincer [23] investigated environmental factors’ effects on African countries’ health indicators. The paper indicated that environmental factors such as carbon emission, urbanization, land under cereal production [24], and water and sanitation facilities effected health indicators of African countries. Countries paid more health expenditures on health services to get fit and healthy.

Engle, Mei [25] calculated direct and indirect carbon emissions from urban and rural households. The input–output method was used to calculate the results for the data set from 1996 to 2012 in China. The causal relationship was found between carbon emissions and rural urban household activities to produce emissions. The study used the causality test and cointegration technique. The study explored the unidirectional relationship of urbanization with direct and indirect carbon emissions from households. Hasan, Bègue [26] explored the relationship of urbanization, energy, and carbon emissions in MENA countries for the time period of 1980–2009. MENA countries are Middle East and North African countries. The study was held to find the effects of urbanization in this region. This study found the bidirectional relation between variables. Urbanization positively impacts carbon emissions and has a positive link with energy use. Panel unit root and Panel Granger causality tests were applied to find the long-run and short-run causal relation among the three variables.

Aydin, Casey [27] conducted the most recent study to access the relationship among buildings and carbon emissions. The study found an increase in carbon emissions with the construction of more buildings. Use of air conditioners, cooling refrigerators, and other electrical appliances to maintain the cleanness of buildings increased with the number of buildings. Building material is another reason to produce more emissions. Nilsson and Brose Olsen [28] studied the health effects of business cycles in 15 Europion Union (EU) countries. Business cycles strongly impact unemployment, affecting mortality rates in return. Unemployment is linked with the earnings of a man and his health level. The study period included the 2008 recession and discussed the results with respect to mortality rates. It reported that at the time of unemployment, mortality rates goes down.

Ilhan and Yobas [29] confirmed the causal relationship of economic and environmental variables of Tunisia for the period of 1971–2012. The ARDL approach was used for long-run relationships among the variables, while the CUSUM test was used for the instability of variables. This study found no support for the environmental Kuznets curve (EKC). York [30] considered 14 EU countries for a period of 40 years. The study estimated the energy consumption of 14 countries from 1960 to 2000 and estimated the future consumption of energy in 2015. The study used demographic factors such as population size and age structure in the area of study. Economic trends were used to predict energy consumption using cross sectional time series data. The study established 14 panels and 41 time periods for this estimation purpose. The study reported that low fertility rates lead to low population size in EU countries. Badulescu, Badulescu [31] found that the environmental Kuznets curve hypothesis is confirmed in most of the European Union economies for the period of 1995−2013. However, deeper analyses at country level displayed different situations and sometimes a negative relation between GDP and government environmental protection.

Payne, Scarborough [32] highlighted that foods with low carbon emissions have better nutritional quality than food having more carbon emissions. He considered dietary items with the permission of the World Health Organization (WHO) and concluded that micronutrients are a key to good health. Shahbaz, Sbia [33] investigated the relationship among CO_2_ emissions and energy intensity per capita in South Africa for 1980–2012. The study used the panel cointegration technique to explore the long-run relationship, for the short-run test it used the ADF unit root test and the Johansen cointegration test. Energy has no significant impact in the short run, while it has long-run impacts in continent. Africa is a coal-rich country and heavily relies on using coal and burning fossil fuels. In burning more fuel and coal, they emit a big share of CO_2_. The environmental Kuznets curve fails in the case of many countries, but it really works in the case of Africa.

Sadorsky [34] presented urbanization’s effects on emerging economies’ carbon emissions. Panel data was collected from 1971 to 2009 for sixteen (16) emerging countries. The study indicated that if the urbanization process has stopped or been controlled, this variable has a lower impact on carbon emissions. The study indicated that charging high taxes to fossil fuel users will reduce emissions and economies can enjoy efficient energy use.

A study on the impacts of urbanization in developing countries was conducted by [35]. The study presented how urbanization affects energy, food system, infrastructure, and the level of production in a given area of study. If the population increases by 10%, it would increase energy consumption from 4.5 to 4.8% of GDP. Aloui, Hammoudeh [36] investigated the relationship between GDP and CO_2_ emissions in the G7 (Canada, Italy, France, Japan, Germany, United Kingdom, and United States of America) countries, excluding Germany. The study used annual time series data from the world development indicator (WDI). Bidirectional causality was found between energy consumption and GDP. A univariate relationship for Canada and no causal relationship existed between variables for France. The U.K., the U.S., and France experienced the neutrality hypothesis. The study concluded that the G7 countries are highly energy dependent countries.

Rafindadi, Yusof [37] studied the relationship between air pollution, fossil fuel energy, and water resources in Asia-Pacific countries. The study used data from 1975 to 2012 published by the [38]. The study used individual analysis as well as cross-sectional analysis to identify results. The study used the panel least square technique and two panel least square regression. This study presented that there was significant relationship between variables, but all variables had some variations with respect to time.

Our study uses two different models for each country. We use mortality rate and life expectancy to estimate cointegration equations and structural breaks. Furthermore, we use comparative analysis and study why the structural breaks occur in mortality and life expectancy in the selected sample countries. The names of the selected countries are China, the U.S., India, Russia, and Japan. Mortality rates under age five are getting attention due to the importance of human capital in all the sectors of an economy. It is rate of death per 1000 lives.

### Research Gap

When we talk about carbon emissions, it means we are concerned about CO_2_ emissions, the most abundant gas from all greenhouse gases. It is colorless, nonpoisonous, and odorless greenhouse gas (GHG). The main sources of carbon emission are human activities and the natural environment. Humans cause CO_2_ by burning fossil fuel, coal, oil, and natural gas. Animals and plants create CO_2_ by the process of respiration and photosynthesis, respectively. Plants absorb CO_2_ in photosynthesis, and the remaining CO_2_ is used in oceans and lakes. The problem occurs when humans burn more fossil fuels and more transportation facilities.

More CO_2_ is produced in the environment to get used by plants and animals. Industrial revolution since 1980 is a strong reason for increased levels of CO_2_ emission. Burning fuel, deforestation, use of cement in building infrastructures, transport vehicles, and the making of furniture play their role in the whole process. As the amount of carbon and energy use is increasing around the globe, the temperature is getting hotter and harsher in different regions. The World Bank Group [38] reported the facts about GHG emissions, which said CO_2_ is the principal gas causing hot climatic changes. Production competition is causing pollutants for health indicators of the globe. The United States Environmental Protection Agency presented that 35% of GHG emissions have increased from 1990 to 2010. The global temperature rise is causing several floods and storms in developing nations and developed nations. Sea level is rising, and glaciers are melting fast. This hot climate is causing mortality which is directly concerned to heat.

Health is a mandatory tool to enjoy the lavish lifestyle and a greater source to get wealthy. Global health indicators taken in this paper are life expectancy at birth, mortality with CO_2_ emissions, and energy use. In need of a relationship between energy consumption, carbon emission, and health variables, a study of China on energy consumption and economic growth has been found by Zhang and Cheng [39] which highlighted that economic growth is independent of both energy consumption and CO_2_ emissions by using the multivariate model of economic growth. For the search of a relation between CO_2_ and health, the study found the with better air quality and reduction of fine-particulate matter in air, life expectancy should increase. Badulescu, Badulescu [31] support the necessity and opportunity of large-scale national and regional policies focused on the beneficial influence of national happiness, healthcare, and environmental expenditure on certain sectors (such as tourism tourism arrivals and receipts) but also on the economy as a whole in the long run.

The current study uses carbon-emitting countries, which are also major users of energy consumption, fossil fuel energy consumption, and also have strong economies. The study uses health indicators defined by millennium development goals to test the structural breaks of health indicators with the relation of CO_2_ emissions and energy consumption in five major emitters. In our research, the main research questions are as follows.

Does a structural break in time series data have some relevance with policy changes or not?

Do CO_2_ emissions and energy consumption have any causal relationship with health indicators?

## 3. Research Methodology

### 3.1. Theoretical Framework

There is no direct relationship between GDP and carbon emissions, as the level of carbon emissions in a country can be influenced by a variety of factors, such as the country’s energy mix, its level of industrialization, and its population size. However, there is generally a positive correlation between GDP and carbon emissions, as countries with higher GDPs tend to have higher levels of industrialization and consume more energy, which can result in higher levels of carbon emissions [40].

There is also no direct relationship between GDP and life expectancy or mortality rate. However, there is generally a positive correlation between GDP and life expectancy, as countries with higher GDPs tend to have higher levels of economic development, which can lead to better health outcomes, including longer life expectancy. Similarly, there is generally a negative correlation between GDP and mortality rate, as countries with higher GDPs tend to have lower mortality rates due to better healthcare, education, and other factors that can contribute to improved health outcomes [41].

Health indicators have a major effect on the economic growth of a country. Life expectancy and health expenditures help to grow GDP. Lower life expectancy is a key variable of poor economic development. Lean and Smyth [42] has assessed the impact of life expectancy on the economic growth of Bangladesh and represented higher economic growth with more life expectancy. The literature on health economics provides evidence that unplanned investment on health caused serious mortality and epidemics in Southeast Asian countries. Approaching the role and effects of economic growth, environmental pollution and noncommunicable diseases on health expenditures, Badulescu, Simut [43] indicated that economic growth was one of the most important factors influencing health expenditures both in the long- and short-run in all the 28 EU countries, during the 2000–2014 period. They also found a negative impact of CO_2_ emission on health expenditure in the short-run, but a positive impact in the long-run. Additionally, the variation in environmental expenditure produces changes in noncommunicable diseases’ effect on health expenditure [43]. Li, Qu [44] presented a strong relationship between health and economics. GDP leads to high health expenditures, in response, better health leads to productivity which increases GDP. Economic activities harm the natural environment as polluted water, land, and air. These environmental issues affect mortality rates by lowering access to resources, food, and clean water. When environmental harm causes more health expenses, health expenditures increase and people suffer from health losses.

### 3.2. Data Collection

This study includes CO_2_ emissions, energy use (kg of oil equivalent per capita), GDP (current US$), fossil fuel (% of total), mortality under age 5, and life expectancy at birth. The study also aims to find a long-run relationship between variables and structural breaks. This study uses energy consumption as a proxy variable (used in cement production) and neglects fossil fuel energy consumption. The study uses two different models each with life expectancy and mortality rate. Time series data were collected from the world development indicators (WDI) for 1975–2015 on mentioned variables. GDP is used as an economic variable. The study further uses health proxies including life expectancy at birth in number of total years and mortality rate under age 5 (millennium development goals).

This study is short on data for Russia. To avoid this problem, the variable generating procedure was used by Stata. First, we generated all the missing values and their logs to make data useful for accurate results. Data were collected from world development indicators (WD1, 2019). To investigate the relationship between energy, CO_2_ emission, and health indicators in carbon-emitting countries, we argued that we could adopt an econometric model of relationship among variables. This study aimed to work on time series data of the top carbon-emitting countries to test the behavior of variables in long- and short-run adjustments. Data are affected by the availability for the Russian Federation.

### 3.3. Explanatory Variables and Expected Signs

Please refer to Table 1 for variables and their expected signs.

**Table 1:** Variables and their expected signs.

Variables	Definition	Expected Sign	
CO_2_ emissions (metric tons per capita)	CO_2_ emission is measured by total amount of CO_2_ emission from production and consumption process divided by population of country.	+	
GDP (current US$)	The gross domestic product and sum of all the production made by producers plus taxes minus subsidies, if any. GDP is calculated at current USD without any deductions for depreciation of product.	+	
Mortality	Mortality rate under age 5 is calculated by number of deaths per 1000 lives.	+	
Life expectancy	Life expectancy at birth is the average number of years a baby born can live, if mortality rates remain constant in coming years.		−
Energy consumption	This is the approximate amount of energy that can be extracted from one kilogram of crude oil.	+	
Fossil fuel energy	It comes from coal, petroleum, natural gas.	+	

### 3.4. Research Model

Study uses time series data to perform research models in Stata for model estimations and equations. All the mentioned variables are endogenous in nature and the study uses a descriptive analysis approach. The study aims to find the relationship between CO_2_ emissions, energy use, and health indicators. The study uses variables with natural logarithms to smooth series. It uses two different models: life expectancy and mortality.

GDP, CO_2_, L_exp_, F_fuel_, Energy.

GDP, CO_2_, Mort, F_fuel_, Energy.

### 3.5. Theoretical Framework

#### 3.5.1. Stationary Test

First, the study used a stationary test to verify integration order. Most of the time series data provides spurious results in long run, if series are not stationary. In the literature, there exist three tests for stationarity, namely, Zivot Andrew (1992), Philip Perron (1997), and augmented Dickey–Fuller test (ADF,). This study used the ADF test for stationary. A nonstationary series poses a unit root if all the series are stationary at their first difference. This study used the ADF test with level and first difference. Most of the series were stationary at first difference.

Test equation

Δyt = α0 + α1 yt − 1+ α2 t + δ1 Δyt − 1 + εt (1)

where α = ρ − 1 and εt is white term.

Hypothesis of study:

**H0:***series are not stationary*.

Δ*Xt* = Γ1Δ*Xt*−1 + ⋯+ Γ*k*−1Δ*Xt*−*k* + ΠΔ*Xt*−*k* + *εt* (2)

Γ*i* = −*I* + Π1 + ⋯+ Π*i*, *i* = 1, (3)

**H1:** *series are stationary.*

#### 3.5.2. Johansen Cointegration Test

This study further uses the cointegration test when all the series are stationary at their first difference. The Johansen test computes multiple cointegrated equations also and this test provides evidence for long-run relationships between variables. It uses trace stat and max stat along with eigenvalue and maximum ranks as likelihood ratios (LR). The Johansen cointegration test with trace value provides the r value and max stat provides the evidence for r+1 maximum rank order. Null and alternate hypothesis are as follows:

**H0**. *There is no long-run relationship/ r ≺ k*.

**H1**. *There is a long-run relationship between variables/r = k*.

If the trace stat is greater than the 5% critical value, the study rejects the null of no long-run relationship. Furthermore, it provides evidence to compute VECM when the series are cointegrated of multiple ranks. There exist cointegration in series, so we do not use the VAR model to test long-run variables’ adjustments. For the long-run adjustments, the study used VECM and it also provides evidence for short-run adjustments.

#### 3.5.3. Vector Error Correction Model (VEM)

After the conclusion of the Johansen cointegration test, we now investigate the long- and short-run time series behavior. This test provides two results for each model. The short-run test is used to estimate the error correction parameters. The long-run test predicts the equilibrium of variables over a long time.

The study further uses diagnostic tests on VEC model to check if the model is stochastic and, hence, if the outcomes of the model are unbiased. The LaGrange-multiplier test was used for residuals autocorrelation. For the purpose of normality study, we used the Jarque–Bera test.

#### 3.5.4. Gregory Henson Structural Break Test

This study used the structural break test of Gregory Henson to find structural breaks of series. It is an extension of the residual base test and includes three test statistics: ADF, zt, and za. This study used these three tests to find breaks in level, level with trend, and regime shift also. Lag length was chosen using the Bayesian information criterion.

##### Specification of Variables

Variables include GDP (gdp), CO_2_ emissions (co), life expectancy (lexp), mortality rate (mort), energy use (enr), and fossil fuel energy consumption (ff).

## 4. Research Results

This section provides evidence of correlation between the variables and the stationary test of the ADF test. Correlation between GDP, CO_2_, ENR, FF, mort, and l_exp_ for China, the U.S., Russia, India, and Japan was calculated separately for each country. Stata was used to compute the comparative analysis of the sample countries. After investigation of the individual analyses of endogenous variables, the study moved towards the stationary test of time series data. The study applied the ADF unit root test to find out if the series is nonstationary. The augmented Dickey–Fuller unit root test let us know whether the variables are integrated of I(1) or I(0). Next, towards cointegration, we used the Johansen cointegration test for the number of integrating equations and long relationship between variables. The Gregory Henson test of structural break was used for break points. 

### 4.1. China: Correlation Matrix of variables

Table 2 shows the correlation matrix of China with the features of life expectancy and life mortality related to the emission of CO_2_, consumption of energy, and the country’s GDP. Please refer Table 2 for correlation results. 

Table 2 includes all the six variables of study for China. Data was collected from 1975 to 2015. The correlation coefficient is the measure of strength between two variables. This study used the Pearson correlation (r). R ranges between −1(perfect -ve relationship) and +1 (perfect +ve relationship). If a value is near to −1 and +1 it shows evidence of strong correlation. The study uses 5% significance level and all the variables with * show linear correlation. The matrix shows GDP has strong +ve correlation with CO_2_, energy, and life expectancy. There is a 0.99% positive correlation between CO_2_ and energy consumption.

#### 4.1.1. U.S.

Table 3 shows the correlation matrix of the U.S. with the features of life expectancy and life mortality related to the emission of CO_2_, consumption of energy, and the country’s GDP. 

Table 4 shows significant results for correlation between variables. A 1% increase in GDP has strong correlation with life expectancy and mortality. There exists weak correlation of energy with mortality and life expectancy.

#### 4.1.2. Russia

Table 4 shows the correlation matrix of Russia with the features of life expectancy and life mortality related to the emission of CO_2_, consumption of energy, and the country’s GDP.

**Table 4:** Correlation matrix of Russia.

	r_gdp	r_lexp	r_mort	r_ff	r_enr	r_co
r_gdp	1.00001					
r_lexp	0.7346 *	1.0000				
r_mort	−0.8896 *	−0.3732 *	1.0000			
r_ff	−0.4198 *	−0.0956 *	0.6105 *	1.0000		
r_enr	0.4880 *	0.5687 *	−0.2293 *	0.2808 *	11.00000	
r_co	0.5655 *	0.4019 *	−0.3537 *	0.0356 *	0.9615 *	1.0000

* shows the level of confidence.

Table 4 shows strong correlation of GDP and life expectancy (0.7346), GDP and mort (−0.8896), and enr and CO_2_ (0.9615)

#### 4.1.3. India

Table 5 shows the correlation matrix of India with the features of life expectancy and life mortality related to the emission of CO_2_, consumption of energy, and the country’s GDP. 

Table 5 shows a strong correlation of selected variables of India. All the variables are strongly correlated with each other. A 1% increase in GDP leads to a 0.96% increase in life expectancy.

#### 4.1.4. Japan

Table 6 shows the correlation matrix of Japan with the features of life expectancy and life mortality related to the emission of CO_2_, consumption of energy, and the country’s GDP.

### 4.2. Stationary Tests: ADF Test of Stationarity

Please refer Table 7 for ADF test.

#### Hypothesis Testing:

**H0**. *There is unit root in series*.

**H1**. *There is no unit root in series*.

**Table 7:** Augmented Dickey–Fuller (ADF) and Zivot-Andrews unit root test for all of the selected countries.

Augmented Dickey–Fuller Test (ADF)				Zivot-Andrews Unit Root Test
Countries	Variables	Level	Difference	Structural Break Year
China	Gdp	−1.681	−3.750 *	2009
	Lexp	−9.159 *	−4.001 *	1986
	Mort	−5.403 *	−2.687	2008
	Ffuel	−3.536	−4.323 *	2003
	Ener cons	−1.720	−3.021 *	2003
	CO_2_	−2.635	−3.419 **	2003
U.S.	Gdp	−2.711	-3.879 *	2003
	Lexp	−2.814	−4.323 *	2004
	Mort	−1.611	−6.511 *	1990
	Ffuel	−1.993	−4.248 *	1996
	Ener cons	−2.013	−4.353 *	2008
	CO_2_	−2.022	−4.341 *	2008
Russia	Gdp	−1.854	−5.445 *	1996
	Lexp	−1839	−3.734 *	1992
	Mort	−2.668	−3.883 *	1989
	Ffuel	−0.418	−5.419 *	2009
	Ener cons	−4.017 *	−2.083	1998
	CO_2_	−4.621 *	−3.275 **	2010
India	Gdp	−1.296	−3.320 **	2009
	Lexp	0.512	−9.081 *	1994
	Mort	−0.077	−9.694 *	1997
	Ffuel	−0.339	−3.631 *	1985
	Ener cons	0.648	−3.298 **	2009
	CO_2_	−1.809	−8.146 *	2008
Japan	Gdp	−1.440	−4.185 *	1986
	Lexp	−2.038	−6.131 *	2008
	Mort	−1.833	−3.542 *	1993
	Ffuel	−1.361	−5.607 *	2009
	Ener cons	0.312	−4.164 *	1988
	CO_2_	−1.917	−4.453 *	1990

Significance level indicates: ** for 5%, * for 10%.

The ADF (augmented Dickey–Fuller) test results presented in the table show that except for mort and co2, all the other variables are stationary at their first difference. The ADF test has evidence that all the series have a unit root inside. Series with unit roots become stationary with l(1). All the series are significant at 5% and 10% levels of confidence. ADF is being used in this study to find the stationarity of the time series data for all the variables of study. We have run the ADF test at level and first difference, and only four series are stationary at level with 95% confidence interval. All the other series are stationary at the difference. China’s mortality rate is fixed at second difference with a −6.511 test statistic. Russia’s GDP and mortality rate are series at second and third difference, respectively. India’s mortality rate is also stationary at third difference. This study finds strong evidence of a cointegration relationship. The ADF tests suggest to apply cointegration for multiple integration equations in selected series.

### 4.3. Johansen Test of Cointegration with Trace Value and Max Value/Cointegration Test (Rank test)

Hypothesis creation criteria:

**H0**. *There is no cointegration*.

**H1**. *There is cointegration*.

The Johansen cointegration test results are presented in Table 8. It follows trace stat and eigenvalue with 5% significance level. There are multiple cointegration equations and it indicates that a long-run relationship exists between variables. R shows the number of ranks and the null hypothesis of series. If the value of the trace stat is greater than the critical value, it rejects the null hypothesis of no cointegration. The study for China shows three integrated equations of life expectancy and four integrated equations with mortality. Please refer Table 9 for max statistic.

Max stat values are a little different from trace statistics. One can use any of the above-described tables. 

### 4.4. VECM and Johansen Normalization Test

#### 4.4.1. Johansen Normalization Restriction Imposed: China

As this study has multiple cointegrated equations, we did not use unrestricted VAR (vector autoregressive). The vector error correction method was used for more than one integrated equation, and it computed the unrestricted errors and long-run integration equations. ECM gives the speed adjustment parameters and long-run equation. For this purpose, the study used two different series each with life expectancy and mortality rate. As the Engel and Granger approach is restricted because it is used for a single equation with only one dependent variable, this study used the Johansen cointegration method for multiple equations. Please refer Table 10 for long-run equilibrium equation. 

**Model 1: c_gdp c-lexp c_ff c_enr c_co.**

**Model 2: c_gdp c_mort c_ff c_enr c_co.**

**Table 10:** Long-run equilibrium equation.

Beta	Coef	Std.Err	Z-Stat	*p*-Value
c_gdp	1	.	.	.
c-lexp	−26.71612	3.544041	−7.54	0.000
c_ff	4.055589	2.024396	2.00	0.045
c_enr	−4.808878	0.9985997	−4.82	0.000
c_co	3.014065	0.9449979	3.19	0.001
_cons	99.85754			

This is the normalization restriction result, and the target variable is GDP as it is the dependent variable in our model. To interpret this equation, the study must reverse the signs of variables. Life expectancy and energy have +ve effect on GDP, while fossil fuel and CO_2_ affect GDP. The coefficients are statistically significant at 1% critical value.

**ECT of the cointegration equation and long-run model**

$$\begin{array}{ll} {\mathrm{ECT}}_{t}{{}_{-}}_{1}= & 1.000C-{\mathrm{GDP}}_{t}{{}_{-}}_{1}-26.71612c-{\mathrm{lexp}}_{t}{{}_{-}}_{1}+4.055589c-{\mathrm{ff}}_{t}{{}_{-}}_{1}\\ & \;-4.80889c-{\mathrm{enr}}_{t}{{}_{-}}_{1}+3.014065c-{\mathrm{co}}_{t}{{}_{-}}_{1}+99.85755 \end{array}$$

**VECM cointegration equation**

$$\begin{array}{ll} \Delta c-{\mathrm{gdp}}_{t}= & 0.05367-0.1032435\Delta c-{\mathrm{gdp}}_{t}{{}_{-}}_{1}-5.7214\Delta c-{\mathrm{lexp}}_{t}{}_{1}\\ & \;-3.710818\Delta c-{\mathrm{ff}}_{t}{{}_{-}}_{1}+1.304151\Delta c-{\mathrm{enr}}_{t}{{}_{-}}_{1}+0.4577989\Delta c\\ & \;-{\mathrm{co}}_{t}{{}_{-}}_{1}+0.0340462{\mathrm{ECT}}_{t}{{}_{-}}_{1} \end{array}$$

Δ shows change in GDP.
Constant term is 0.05367.
Here, λ is (ECT) adjustment parameter, and it is 0.03404.

Interpretation: The result shows that the adjustment term (0.034%) is statistically significant at 10% significance level. It suggests that last year’s errors are corrected within this year at a 3.4% speed of convergence. Please refer Table 11, Table 12, Table 13, Table 14 and Table 15 for Johansen normalization.

**Mortality:** This is the normalization restriction equation of mortality. GDP is the dependent variable and the coefficients are statistically significant at 1% critical value. Mortality, fossil fuel, and energy use have an effect on GDP. Co2 has +ve effect on GDP.

#### 4.4.2. Johansen Normalization Restriction Imposed: U.S.

**Model 1: us_gdp us_lexp us_ff us_enr us_co.**

**Model 2: us_gdp us_mort us_ff us_enr us_co.**

**Table 12:** Johansen normalization restriction imposed for U.S.

Beta	Coef	Std.Err	Z-Stat	*p*-Value
us_gdp	1	.	.	.
us-lexp	−28.25588	1.270396	−22.24	0.000
us_ff	15.64236	1.300421	12.03	0.000
us_enr	15.20434	1.994074	7.62	0.000
us_co	−15.27256	1.775366	−8.60	0.000
_cons	−67.60238			
us_gdp	1	.	.	.
us-mort	0.6196919	.5297686	1.17	0.242
us_ff	−19.65899	4.649569	−4.23	0.000
us_enr	−26.45132	6.525074	−4.05	0.000
us_co	25.1588	5.690274	4.42	0.000
_cons	217.5517			

#### 4.4.3. Johansen Normalization Restriction Imposed: Russia

**Model 1: r_gdp r_lexp r_ff r_enr r_co.**

**Model 2: r_gdp r_mort r_ff r_enr r_co.**

**Table 13:** Johansen normalization restriction imposed for Russia.

Beta	Coef	Std.Err	Z-Stat	*p*-Value
r_gdp	1	.	.	.
r-lexp	3.838708	6.989457	0.55	0.583
r_ff	−29.85799	36.438	−0.82	0.413
r_enr	11.95761	19.55452	0.61	0.541
r_co	−31.26217	19.06087	−1.64	0.101
_cons	67.02994	.	.	.
r_gdp	1	.	.	.
r-mort	12.748 *	2.673502	4.77	0.000
r_ff	171.0695	126.1997	1.36	0.175
r_enr	75.29804	58.32422	1.29	0.197
r_co	−28.71538	54.3937	−0.53	0.599
_cons	−1401.393	.	.	.

* shows the level of confidence.

#### 4.4.4. Johansen Normalization Restriction Imposed: India

**Model 1: ind_gdp ind_lexp ind_ff ind_enr ind_co.**

**Model 2: ind_gdp ind_mort ind_ff ind_enr ind_co.**

**Table 14:** Johansen normalization restriction imposed for India.

Beta	Coef	Std.Err	Z-Stat	*p*-Value
ind_gdp	1	.	.	.
ind-lexp	−11.21431 *	1.996255	−5.62	0.000
ind_ff	7.907484 *	0.9008619	8.78	0.000
ind_enr	.5114112	1.052142	0.49	0.627
ind_co	−3.960494 *	1.052142	−3.85	0.000
_cons	−16.8917	.	.	.

* shows the level of confidence.

#### 4.4.5. Johansen Normalization Restriction Imposed: Japan

**Model 1: jp_gdp jp_lexp jp_ff jp_enr jp_co.**

**Model 2: jp_gdp jp_mort jp_ff jp_enr jp_co.**

**Table 15:** Johansen normalization restriction imposed for Japan.

Beta	Coef	Std.Err	Z-Stat	*p*-Value
jp_gdp	1	.	.	.
jp-lexp	−13.73619 *	6.192142	−2.22	0.027
jp_ff	−40.06873 *	11.57994	−3.46	0.001
jp_enr	−43.50596 *	12.10509	−3.59	0.000
jp_co	46.55649 *	12.49621	3.73	0.000
_cons	463.0919 *	.	.	.

* shows the level of confidence.

#### 4.4.6. Gregory Henson Structural Break Test

There are different tests for structural breaks such as chow test, sup-Wald, sup-LM, sup-LR, and Hatemi-J test. This study used the Hansen [45] for one unknown structural break test. The study has two different models for the Gregory Henson test of cointegration. Maximum lag length is chosen using the Bayesian information criterion (BIC). The study used break in level, break in trend, and break in regime for two models of each country. ADF, zt, and za test statistics were used with 1%, 5%, and 10% critical values. The study used ADF test statistics as it provided the lowest test at 95% confidence interval. Please refer Table 16 for Gregory Henson structural break test.

**Model 1 includes GDP, lexp, co2, and energy consumption.**

**Model 2 includes GDP, mort, co2, and energy consumption.**

**H0**. *There is no cointegration at the break point.*

**H1**. *There is cointegration at the break point.*

**Table 16:** Gregory Henson structural break test for all five countries.

Countries		t-Stat	Level (lags)	t-Stat	Trend (lags)	t-Stat	Regime (lags)
China	Lexp	−4.00 *	1992(0)	−4.22 *	1987(0)	−4.10 *	2002(0)
	Mort	−4.06 *	1992(0)	−4.27 *	1980(0)	−4.44 *	1991(1)
U.S.	Lexp	−5.49 *	1980(0)	−5.40 *	1980(1)	−6.20 *	1981(0)
	Mort	−3.32 *	2001(1)	−3.62 *	1983(2)1993	−3.15 *	1993(0)
Russia	Lexp	−3.32 *	1985(0)	−3.05 *	1985(0)	−3.33 *	1990(2)1985
	Mort	−3.43 *	1979(0)	−3.57 *	1991(0)	−4.20 *	1991(0)1980
India	Lexp	−3.53 **	2005(0)	−3.69 **	2005(0)	−3.94 *	2004(0)
	Mort	−3.80 **	1989(1)	−3.56 **	1989(2)	−4.51 *	2004(0)
Japan	Lexp	−4.39 **	1997(1)	−4.24 **	1998(1)	−4.62 **	1996(2)
	Mort	−4.53 **	1998(1)	−4.55 **	1998(1)	−4.53 **	1996(1)

Note: ADF */zt **/za **

There were changes in health policies that were implemented in China in 1992 as part of the “Healthy China 2020” initiative. This initiative aimed to reform the healthcare system and improve public health in China. Some of the changes that were implemented as part of this initiative included the establishment of a basic medical insurance system, the strengthening of primary healthcare services, and the improvement of disease prevention and control measures. These changes may have had an impact on mortality and life expectancy in China [46].

There have been a number of policy changes related to health in the United States over the years, and these may have had an impact on mortality and life expectancy. In 1980, the U.S. government implemented the “Healthy People” initiative, which aimed to improve the overall health of the population through the promotion of healthy behaviors and the prevention of chronic diseases. This initiative may have contributed to improvements in mortality and life expectancy in the 1980s. In 1985, the U.S. government passed the Emergency Medical Treatment and Active Labor Act (EMTALA), which requires hospitals to provide medical care to patients in emergency situations, regardless of their ability to pay. This act may have had an impact on mortality rates, particularly among low-income individuals who previously may not have had access to emergency medical care. In 2001, the U.S. government passed the “Patients’ Bill of Rights,” which aimed to improve the quality of healthcare and protect the rights of patients. This legislation may have had an impact on mortality and life expectancy by improving the overall quality of healthcare in the United States [47].

Russia underwent significant political and economic changes in the late 1980s and early 1990s, which may have had an impact on mortality and life expectancy in the country. In 1979, the Soviet Union implemented a number of health reforms as part of the “Health for All” initiative, which aimed to improve the accessibility and quality of healthcare in the country. These reforms included the expansion of primary care services, the establishment of a network of outpatient clinics, and the introduction of new technologies and treatments. These changes may have contributed to improvements in mortality and life expectancy in Russia in the 1980s. In 1985, the Soviet Union adopted a new policy of “glasnost,” or openness, which led to greater political and economic openness and a relaxation of government control. This policy may have had an impact on mortality and life expectancy by improving living conditions and access to resources for the population [48].

In 1989, India had a mortality rate of 30.9 deaths per 1000 population and a life expectancy of 60.3 years. By 2005, there was a significant improvement in both mortality and life expectancy in India. The mortality rate had decreased to 21.3 deaths per 1000 population and the life expectancy had increased to 65.3 years.

This improvement can be attributed to several factors, including increased access to healthcare, improved healthcare infrastructure, and increased awareness about health and disease prevention. The government also implemented various policies and programs aimed at improving the health and well-being of its citizens, such as the National Health Mission and the National Rural Health Mission [49].

In 1997, Japan had a mortality rate of 8.2 deaths per 1000 population and a life expectancy of 79.6 years. In 1998, there was a slight improvement in both mortality and life expectancy in Japan. The mortality rate decreased to 8.1 deaths per 1000 population and the life expectancy increased to 79.8 years. This improvement can be attributed to several factors, including Japan’s strong healthcare system and high levels of access to medical care. The country also has a low infant mortality rate and a high rate of vaccination coverage which contribute to overall improved health outcomes. Additionally, Japan has implemented various policies and programs aimed at improving the health and well-being of its citizens, such as the National Health Insurance system, which provides universal healthcare coverage for all citizens. The government has also prioritized preventative health measures, such as promoting physical activity and healthy eating habits, which have likely contributed to the slight improvement in mortality and life expectancy in 1997 and 1998 [50]. Please refer Table 17 for carbon-emitting countries. 

## 5. Conclusions and Policy Implications and Limitations

The primary objective of this paper was to investigate the relationship between energy use, economic growth, and CO_2_ emissions between 1965 and 2015 in developed countries. The augmented Dickey–Fuller (ADF) and some other tests were used to verify the stationary existence of each time sequence. The augmented Dickey–Fuller (ADF) and Phillips–Perron (PP) unit root test results suggested that all-time series are stationary at the level and first difference and there is no second difference in either of the series. Autoregressive distributed lag (ARDL) was applied to control cointegration between the sequences. Long-term results from ARDL pointed out that energy consumption in developed countries has a positive effect on CO_2_ emissions. The coefficient of economic growth indicated a positive short-term and long-term impact on CO_2_ emissions in developed countries.

This paper’s major goal was to analyze the connection between energy use, GDP growth, and CO_2_ emissions in LDCs between 1965 and 2015. The time series were tested for stationarity using the Phillips–Perron (PP) and augmented Dickey–Fuller (ADF) unit root tests. All-time series were level and first difference stationary, and none were at the second difference, as determined by the augmented Dickey–Fuller (ADF) and Phillips–Perron (PP) unit root tests. Cointegration among the series was tested using autoregressive distributed lag (ARDL). The results from ARDL showed that long-term energy use had a favorable influence on CO_2_ emissions in developing nations. Long-term and short-term effects of economic expansion on CO_2_ emissions in developing countries are both favorable, according to the coefficient.

### Policy Implications and Limitations

This study’s findings suggest that to slow environmental deterioration in developing nations, governments should promote forest-related education and training for locals in cooperation with their respective forest departments. Based on these estimates, it is proposed that developing nations adjust their economic growth policies to better regulate environmental degradation. It is advised that developing nations choose energy sources that generate the least amount of environmental damage, as nonrenewable sources are widely used for fuel, industrial production, and residential energy consumption. Policymakers are urged to adopt measures that promote the use of environmentally friendly equipment, machinery, vehicles, and utilities if they are serious about preventing and reversing environmental damage over the long term.

Based on the results of this report, it is recommended that developed countries’ governments should educate local people to enable them to prepare with the Department of Forests to increase the proportion of forests in developed countries to monitor environmental degradation. The projected results show that environmental degradation is the key cause of economic growth, so it is recommended that developed countries’ economic growth strategies be revised in order to monitor environmental degradation. In developed countries, nonrenewable energy sources are used as fuel and for industrial production and household energy consumption, so it is recommended that certain sources of energy be adopted that cause minimal environmental degradation. Policymakers are urged to implement certain policies that promote the use of environmentally friendly equipment, machinery, automobiles, and services to reduce environmental degradation in order to monitor environmental degradation in the long term.

## Figures and Tables

**Table 2:** Correlation matrix of China.

	c_gdp	c_lexp	c_mort	c_ff	c_enr	c_co
c_gdp	1.0000					
c_lexp	0.9672 *	1.0000				
c_mort	−0.9841 *	−0.9585 *	1.0000			
c_ff	0.9605 *	0.9801 *	−0.9319 *	1.0000		
c_enr	0.9859 *	0.9505 *	−0.9913 *	0.9467 *	1.0000	
c_co	0.9872 *	0.9689 *	−0.9820 *	0.9689 *	0.9940 *	1.0000

* shows the level of confidence.

**Table 3:** Correlation matrix of the U.S..

	us_gdp	us_lexp	us_mort	us_ff	us_enr	us_co
us_gdp	1.0000					
us_lexp	0.9848 *	1.0000				
us_mort	−0.9972 *	−0.9805 *	1.0000			
us_ff	−0.9474 *	−0.9161 *	0.9457 *	1.0000		
us_enr	−0.5075 *	−0.5723 *	0.4940 *	0.5930 *	1.0000	
us_co	−0.6989 *	−0.7495 *	0.6882 *	0.7720 *	0.9630 *	1.0000

* shows the level of confidence.

**Table 5:** Correlation matrix of India.

	ind_gdp	ind_lexp	ind_mort	ind_ff	ind_enr	ind_co
ind_gdp	1.0000					
ind_lexp	0.9698 *	1.0000				
ind_mort	−0.9897 *	−0.9834 *	1.0000			
ind_ff	0.9230 *	0.9827 *	−0.9418 *	1.0000		
ind_enr	0.9833 *	0.9709 *	−0.9949 *	0.9315 *	1.0000	
ind_co	0.9715 *	0.9912 *	−0.9874 *	0.9754 *	0.9867 *	1.0000

* shows the level of confidence.

**Table 6:** Correlation matrix of Japan.

	jp_gdp	jp_lexp	jp_mort	jp_ff	jp_enr	jp_co
jp_gdp	1.0000					
jp_lexp	0.9047 *	1.0000				
jp_mort	−0.9035 *	−0.9955 *	1.0000			
jp_ff	−0.5886 *	−0.3621 *	0.3592 *	1.0000		
jp_enr	0.8883 *	0.7973 *	−0.7729 *	−0.7081 *	1.0000	
jp_co	0.8147 *	0.8284 *	−0.7975 *	−0.3464 *	0.9027 *	1.0000

The correlation matrix of Japan shows a strong correlation of mort, lexp, enr, and CO_2_. * shows the level of confidence.

**Table 8:** Eigenvalue table for all five countries.

Countries	Variables	Rank	Eigenvalue	Trace Stat	Critical Value 5%
China	Lexp	3	0.52225	10.4840 *	15.41
	Mort	4	0.31405	2.0697 *	3.76
U.S.	Lexp	2	0.53329	25.9715 *	29.68
	Mort	3	0.36602	15.3609 *	15.41
Russia	Lexp	3	0.63374	13.2484 *	15.41
	Mort	2	0.72007	23.6083 *	29.68
India	Lexp	4	0.38982	0.6910 *	3.76
	Mort	1	0.66230	43.5124 *	47.21
Japan	Lexp	2	0.42629	26.2232 *	29.68
	Mort	4	0.29571	2.0478 *	3.76

* shows the level of confidence.

**Table 9:** Max statistic table for all five countries.

Countries	Variables	Rank	Eigenvalue	Max Stat	Critical Value 5%
China	Lexp	3	0.52225	8.5078	14.07
	Mort	4	0.31405	2.0697	3.76
U.S.	Lexp	2	0.53329	14.1369	20.97
	Mort	3	0.36602	12.3287	14.07
Russia	Lexp	3	0.63374	9.0887	14.07
	Mort	2	0.72007	13.4297	20.97
India	Lexp	4	0.38982	0.6910	3.76
	Mort	1	0.66230	25.0303	27.07
Japan	Lexp	2	0.42629	14.6433	20.917
	Mort	4	0.2957	2.0478	3.76

**Table 11:** Johansen normalization restriction imposed for China.

Beta	Coef	Std.Err	Z-Stat	*p*-Value
c_gdp	1	.	.	.
c-mort	3.240817	0.6100383	5.31	0.000
c_ff	20.25925	3.392361	5.97	0.000
c_enr	10.02829	2.273876	4.41	0.000
c_co	−9.716399	1.93303	−5.03	0.000
_cons	−183.4108	.	.	.

**Table 17:** Top 5 carbon-emitting countries, 2018.

Countries	CO_2_ Emissions 2018 (Billion Metric Tons)	Global Share
China	9.43	27.8%
U.S.	5.15	15.2%
Russia	2.48	7.3%
India	1.55	4.6%
Japan	1.15	3.4%

Source: world development indicators.

## Data Availability

Data will be provided on demand from corresponding author.
